# Demystifying Supervised Learning in Healthcare 4.0: A New Reality of Transforming Diagnostic Medicine

**DOI:** 10.3390/diagnostics12102549

**Published:** 2022-10-20

**Authors:** Sudipta Roy, Tanushree Meena, Se-Jung Lim

**Affiliations:** 1 Artificial Intelligence & Data Science, Jio Institute, Navi Mumbai 410206, India; 2 Division of Convergence, Honam University, 120, Honamdae-gil, Gwangsan-gu, Gwangju 62399, Korea

**Keywords:** healthcare, artificial intelligence, supervised learning, computer vision, medical imaging, deep learning, precision medicine, XAI

## Abstract

The global healthcare sector continues to grow rapidly and is reflected as one of the fastest-growing sectors in the fourth industrial revolution (4.0). The majority of the healthcare industry still uses labor-intensive, time-consuming, and error-prone traditional, manual, and manpower-based methods. This review addresses the current paradigm, the potential for new scientific discoveries, the technological state of preparation, the potential for supervised machine learning (SML) prospects in various healthcare sectors, and ethical issues. The effectiveness and potential for innovation of disease diagnosis, personalized medicine, clinical trials, non-invasive image analysis, drug discovery, patient care services, remote patient monitoring, hospital data, and nanotechnology in various learning-based automation in healthcare along with the requirement for explainable artificial intelligence (AI) in healthcare are evaluated. In order to understand the potential architecture of non-invasive treatment, a thorough study of medical imaging analysis from a technical point of view is presented. This study also represents new thinking and developments that will push the boundaries and increase the opportunity for healthcare through AI and SML in the near future. Nowadays, SML-based applications require a lot of data quality awareness as healthcare is data-heavy, and knowledge management is paramount. Nowadays, SML in biomedical and healthcare developments needs skills, quality data consciousness for data-intensive study, and a knowledge-centric health management system. As a result, the merits, demerits, and precautions need to take ethics and the other effects of AI and SML into consideration. The overall insight in this paper will help researchers in academia and industry to understand and address the future research that needs to be discussed on SML in the healthcare and biomedical sectors.

## 1. Introduction 

**Figure 1:**
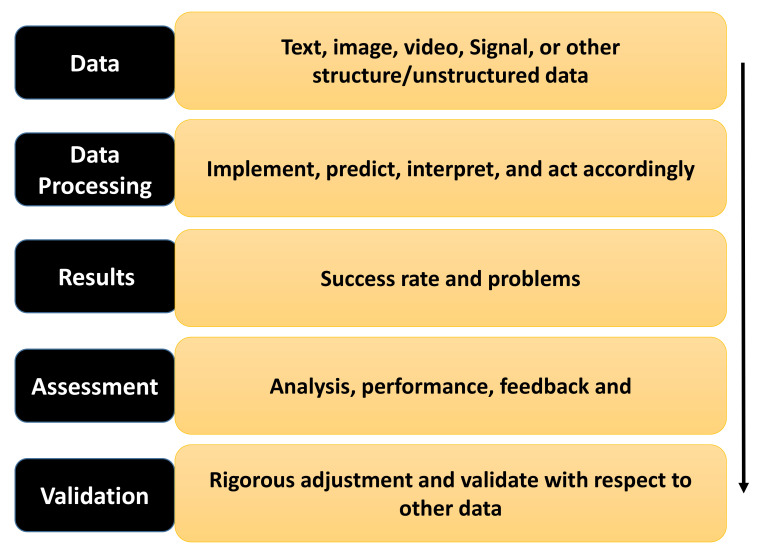
Working flow of SML process in healthcare.

Recent years have seen much clinical and preclinical healthcare research using SML and many other AI techniques. More specifically, SML has changed almost all sectors globally in terms of digital healthcare, such as accurate disease detection and classification. Many academic labs and industries are working to develop AI tools for different healthcare areas [1]. SML can deliver better support to the medical practitioner and assist them in making better clinical decisions [2]. SML has the self-learning capabilities to enhance accuracy. The SML technique also uses advanced methods to foresee health risks and generate warnings by extracting meaningful features from structured and unstructured large data. Currently, SML has extended to more accurate methods with the increased use of an advanced form of machine learning (ML), deep neural network (DNN)/deep learning (DL), natural language processing (NLP), computer vision (CV), robotics, and many other fields. [3]. A brief structure of the SML workflow process of data processing, assessment and outcome, and validation in health is shown in Figure 1. 

In many co-clinical research labs, modern SML techniques are used to predict and diagnose risky diseases such as cancer and neuronal and cardiac disorders, where timely detection and analysis are super important [4]. The product-based deployment of SML in biomedical and healthcare industries is also working to deliver an effective and operative environment. Earlier, IBM Watson brought a foremost and fundamental revolution to pioneering SML in the healthcare informatics industry [5].

**Figure 2:**
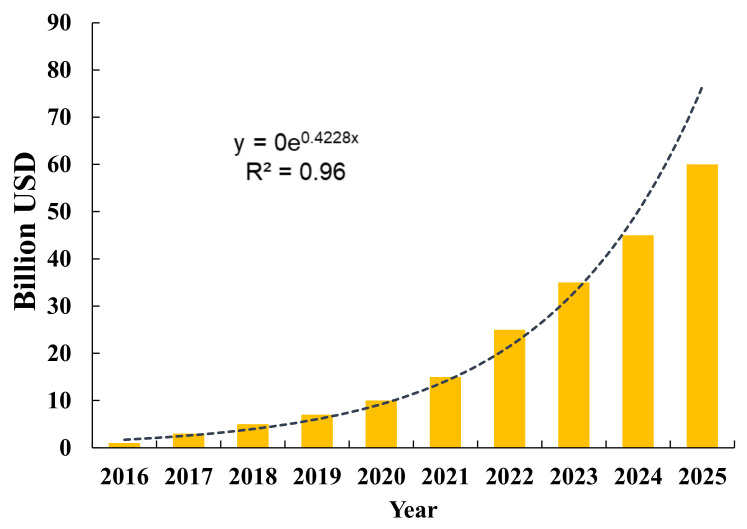
The probable trend of revenue generation trend in the health and biomedical sectors.

The advanced and rigorous SML method can quickly learn patterns and features from bulk-size data and then predict and interpret those complex data to quickly help co-clinical researchers and users. SML is prepared with learning and self-learning capabilities to increase accuracy based on feedback [4]. An SML method also helps to increase analytical and therapeutic accuracy and reduce errors which are practically unavoidable by experts. Furthermore, an SML-based method will be very suitable to assist medical practitioners in making real-time interpretations of health hazards and sending AI-based alerts to the caregiver of the patients. As per the statistical data available [6], the revenue generated from AI in healthcare [6] is shown in Figure 2. 

The primary objective of this paper is to provide an overview of different SML methods, a subsection of AI-based automated healthcare systems for academia and industry 4.0. This will help other researchers in taking the decision to analyze data and improve the accuracy and efficiency involved in biomedical and healthcare data analysis [7,8]. Simultaneously, a new direction for SML in health application program interface (API) development and building is introduced, which will accelerate research on self-learning, decision-making, intellectual behavior, and other issues encountered during implementation, as well as from a data access and security point of view. According to the scientific community, SML in biomedical and healthcare would be the most adjustable and trustworthy technique after a few years. The goal for healthcare is to become more personal, predictive, preventative, and participatory, and AI can make major contributions in all academic, research, and industry directions. We also discuss the concerns regarding AI in the health sector and the possible ways to overcome those problems. From an overview of the progress made, we estimate that AI will continue its momentum to develop and mature as a powerful tool for healthcare sectors.

### 1.1. The Major Data Types Analyzed

The clinical and preclinical data mainly exist in the form of images, videos, signal, raw data, demographics, medical notes, and electronic records from scanners, devices, health check-ups, and laboratories. A considerable amount of SML-based data analyses is derived from diagnostic imaging, genomics, and electro diagnosis [4]. 

**Figure 3:**
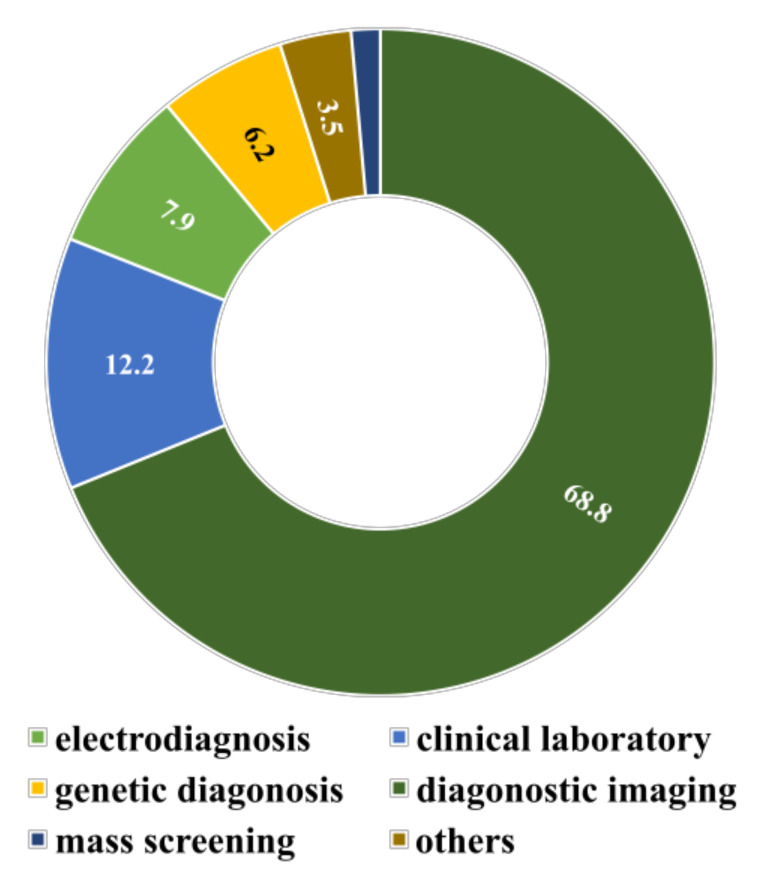
Major data types for supervised machine learning in healthcare research.

The physical prescription of a doctor and laboratory report observation can also be considered two additional data sources. We differentiate them with images, videos, electronic health records (EHR), and genomics from the significant percentages of unstructured data; those unstructured data are used for indirect analysis. Therefore, the corresponding unstructured data analysis and AI/ML can emphasize converting the unstructured data to structured data. The major data types used in SML and their corresponding application percentage [4] are displayed in Figure 3. 

### 1.2. Major Disease Types Currently Tackled by SML 

Although AI is rapidly growing in health sectors, the academic research and development community mainly focuses on a limited number of diseases, such as cardiology, neurology, cancer, and skin-related diseases [4]. A few examples are mentioned below.

1. Cancer: The IBM Watson for oncology was developed using AI to support automated cancer analysis, and a validation study [9] was performed blindly. Skin cancer and its subtypes were identified from clinical images with high accuracy by Esteva et al. [10] who failed to generalize the method.
2. Neurology: Movement of the cortical controller and its restoration in a human using SML was implemented with limited accuracy in patients with quadriplegia [11]. Later, a human and machine interface was created based on release time after re-innervation of the target muscle spinal motor to regulate the prostheses' upper limb [12].
3. Cardiology: Heart disease diagnosis from cardiac images is a great application in SML. Arterys received authorization from Food and Drug Administration (FDA)-USA to market their cardio deep learning (DL) method that provides computerized, editable segmentations on cardiac MRI images [13].

These three diseases are very dangerous and must be acted on at a very early stage. Therefore, timely identification and recognition of a particular kind of disease and treatment are crucial to avoid the further degradation of patients’ health. 

### 1.3. Long-Term Prospect 

SML helps to simplify the future of healthcare sectors and ML-augmented care, precision medicine, diagnostic radiography, precision diagnostics, and medicines, enabling biomedical and healthcare analytics systems to achieve their multiple objectives [14]. ML continues to accelerate rapidly across physical and mental health, including online healthcare consultation, prognosis, disease diagnosis, drug discovery management, and health monitoring. Currently, the SML system is not used the same way as a human medical practitioner. Those can sometimes use their clinical experience and intuition. Today, SML-based systems are started to automate the traditional biomedical task to avoid time-consuming, big complex data, and tedious tasks. SML can also translate patterns and interpret diseases from large-volume datasets. Despite this, there is room for significant improvement in SML regarding precision diagnostics from medical imaging, particularly in diabetic retinopathy (DR) planning and treatment. 

Medium-term (next 10 years): There will be significant improvement and development occurring after the implementation of the most powerful deep learning algorithms for various applications that are efficient, can handle more data, automatic annotation, multiple omics data, handle structure and unstructured data, able to use more unlabeled data, complex 3D and 4D images, complex signals, unstructured electronic health data, and behavioral and pharmacological data. AI/ML will help store and structurally analyze the healthcare sector’s data within the organization. Health check-up practices will change and adopt new technology to structurally analyze the healthcare sector’s data within the organization. These will expand SML applications for personalized and precision medicine and therapeutics study. 

Long-term (beyond 10 years): Considering the long-term prospect, SML in the biomedical and healthcare sector will be fully automatic and more intelligent using a thorough augmented and connected healthcare system. All healthcare and biomedical systems will shift from traditional to SML biomedicine to provide a preventative, more secure, and data-driven personalized disease managing model to achieve an improved patient care system, clinical experiences, and outcomes in a more cost-effective way.

## 2. Growing Research Areas 

### 2.1. Connected/Augmented Care 

AI/ML considerably reduces healthcare inadequacy, escalates patient flow and knowledge, and improves the caregiver’s clinical experience and patient safety care pathway. Automation in healthcare could also be applied to remote monitoring and the use of clinical patients through smartphones, wearable sensors, and IOTs to identify, prevent, and provide timely monitoring of patients [14]. This continuous monitoring will also help to classify high-risk patients using advanced classification techniques. In the long term, we can imagine SML-based healthcare clinics, automated hospital systems, fully automatic personalized social care services, and personalized patients and caregivers connected to a single, interoperable digital infrastructure combined with ambient intelligence. The following are two SML applications in connected care.

#### 2.1.1. AI-Based Chatbots 

Virtual SML Chatbots and recommendation engines such as Babylon for sick care and recommendations and Ada support for health condition prediction with clinical superiority using artificial intelligence are used by many doctors and patients to recognize diseases based on symptoms. SML virtual Chatbots can also be integrated with other imaging tests and wearables to deliver understanding to users and caregivers to improve patients’ wellness.

#### 2.1.2. Ambient and Intelligent Care 

The automation of ambient clinical intelligence leveraging sensors and NLP skill and knowledge can computerize the whole back end of healthcare tasks such as documenting patient’s information in the form of EHR, upgrading and revamping the clinical roadmap, and allowing the medical practitioner to concentrate more time on the patient’s care. Recent research has explored the capability to use smart contact-less devices to observe heart pulses [15].

#### 2.1.3. Digital Consultation

In the COVID-19 era, the use of digital consultation has increased significantly. The idea of digital consultation is to decrease unnecessary hospital visits for negligible warning. Minor cases can be self-treated with special home care and the assistance of a medical consultant without unnecessary wasting of waiting time. The SML-based model provides consultation based on the past medical records of patients, questionaries’ (e.g., Chatbot), and overall information associated with health information and the medical field [16].

Digital consultation is very useful for those patients who have tight work schedules and find it difficult to find time to visit a doctor often or have regular check-ups or treatment for themselves or their relatives and sometimes go for over-the-counter medicines. That is why the SML-based Chabot application, such as Buoy, was introduced in the past and became very popular. Buoy also used some pre-planned replies to the patients [17]. The users can choose doctors depending on their illness and health problems from the options provided in the healthcare digital consultation app.

#### 2.1.4. Internet of Things for Patient Monitoring

The demand for sensors and ML-based automatic progressive modeling for patient monitoring [18] is increasing daily. Many wearables, such as glucometers, blood pressure monitors, oximeters, and many more advanced AI/ML sensor applications, such as smart transplants and prosthetics sensors, are often used in post-surgery patient observation [19]. These AI/ML sensors are used to avoid difficulties after surgery by constantly monitoring important patient health parameters. A new movement of remote patient care systems in the research community, such as smart fabrics, nanorobots, and digital pills, support medication adherence, monitor cardiac disease, and assist in wound supervision. 

### 2.2. Precision Diagnostics

#### 2.2.1. Analytic Imaging 

The automatic detection, segmentation, and classification of co-clinical biomedical images are the foremost SML in the healthcare sector today [14]. Many recent studies [20,21,22,23] have recognized the capability of SML, particularly in radiological medical images that surpass the accuracy of human experts. Examples are as follows: A convolutional neural network (CNNs) DL-based automated pneumonia detection method for chest X-rays overtook the performance of radiologists [20], and another CNN-based DL method was established to classify different skin lesions very accurately [21] to help dermatologists. Lymph node metastases of breast cancer were also accurately detected from whole slide pathology using SML-based methods compared to multiple pathologists [22], and the accuracy of heart attack diagnosis using the complete automatic DL method has surpassed that of cardiologists [23]. 

#### 2.2.2. Diabetic Retinopathy 

Numerous vision-related issues arise due to diabetic retinopathy, and personalized SML DL-based methods are being developed for proper disease recognition and quick treatment plans [24]. Deep learning makes diabetic detection easier because manual one-on-one screening by human observers is expensive due to an increase in the number of diabetes patients worldwide and an insufficient number of eye care professionals. As a result, research and development on computerized diabetic retinopathy detection are increasing in the United States, the Europe Union, the United Kingdom, Singapore, China, and India. A few of them have already cost-effectively validated vigorous diabetic diagnostics [25]. 

#### 2.2.3. Faster Results with More Precision and Accuracy

Many significant SML-based methods are being used to assist medical practitioners in image-based analysis and planning as a non-invasive strategy for cancer treatment and planning [26]. Tasks such as automatic segmentation of target lesions, annotations, and self-learning technology reduce the time and laborious efforts taken by humans using specially designed SML-based methods [27]. Some studies show that the manual preparation and analysis time for head, neck, and prostate cancer is 90 percent higher than when using an SML/ML-based method, and the average waiting time for a radiotherapist’s diagnosis and corresponding procedure can be reduced by at least 50% using an SML-based method [28].

#### 2.2.4. Precision Therapeutics

Researchers all over the world are attempting to explore the cellular and molecular roots of various illnesses. The main challenge is collecting and gathering a diverse set of heterogeneous datasets that can lead to faster implementation of the digital image to biological linking and precision therapeutics; however, these precision therapeutics are still underdeveloped in terms of SML-based method implementation due to small labs, unorganized data, and complex personalized procedures. Considering the prevailing demands of precision therapeutics, synthetic biology and drug discovery could be two promising futures for SML applications.

#### 2.2.5. Immunomics and Synthetic Biology

SML-based methods must be updated and upgraded [29] for a better understanding of the cellular foundation of disease. Along with syntactic biology, the categorical heat map of patient inhabitants’ study can also help to deliver more target-based pre-emptive approaches in immunomics. Although the application of SML tools on multimodal datasets in immunomics and synthetic biology is not progressing yet, this research is still the future. Using immunomics and synthetic biology to diagnose disease will be ground-breaking due to the implementation of special regulators in cancer diagnosis, neurology, and other rare or uncommon disease cases for individual personalized care. 

#### 2.2.6. Cancer Diagnosis

SML is widely applied for classification, detection, and diagnostics in clinical and preclinical cancer studies. Ichimasa et al. reported the usefulness of SML in operations after the resection of T1 colorectal cancer [30]. In clinical practice, the guessing/prediction or identification of any cancer and its aggressiveness (for example, identification of lymph node metastasis) is very difficult, and it is challenging to make a decision towards surgery. An SML-based accurate prediction model can predict and reduce image scanning time. Hence, an SML/ML-based model to analyze lymph node metastasis was implemented on pathological results that can also adopt new information on metastasis to avoid unnecessary surgeries [31]. In another study [32], a deep CNN was implemented to study the transformations and characteristics of non-small cell lung carcinoma from histopathological slides. Still, the method was not interpretable and self-explainable. Their study shows a reduction in patient visits to the hospital as the SML method performed the radiological nodules’ classification work. Various non-Hodgkin lymphomas can be distinguished using deep learning techniques based on outcomes of discriminant studies [32] of lymphoma features. Over the years, lymph-node metastases in breast cancer patients have been successfully detected using deep-learning algorithms from pathology. The early recognition and prognostic assessment in precision oncology using the advanced ML method is also conceivable from pathology. Companies such as IBM, Queri.ai, PAIGE.AI, Inspirata, Proscia, deepMinds, DeepLens, and PathAI use AI/ML tools for the analysis of different cancers and their subtypes.

### 2.3. Drug Discovery 

Significant improvement is possible in clinical and preclinical trial strategy, workout plans, and optimization of overall drug discovery and effectiveness procedures. Particularly in co-clinical study, any combinational optimization technique in the SML-based therapy and drug discovery system could be replaced [33]. Currently, DeepMind and AlphaFold are setting up research and development labs and investments to understand the healthier sympathetic sequence and procedures and predict the structures of protein and protein-to-protein interaction to develop more target-based therapeutics for clinical aspects [34].

Selecting a novel and effective drug from a bunch of potential pharmacological vigorous chemical bodies is a challenging task [35]. SML-based computer-aided drug discovery tools, with the help of clinical researchers, can help medical professionals make decisions in a short amount of time, whereas traditional methods require many years to execute. SML also helps to identify some policies in drug molecule profiling and design by understanding disease profiles from a complex data set. Another SML usage in drug discovery is recognizing the cardiotoxic and nontoxic drugs that belong to the anticancer class. SML can also identify potential antibiotics from millions of molecules, where Halicin is the first antibiotic recognized by SML [36]. These algorithms can also be used to detect molecules [36]. SML can be implemented to distinguishand combat the antimicrobial resistance of molecules and accountable deoxyribonucleic acid sequences for antibiotic resistance. Currently, SML is being used by different academics and industries in many drug discoveries: a team from Toronto University and IBM working together on Ebola virus infections; Sumitomo Dainippon Pharma and Exscientia strategies working on an innovative drug discovery SML method for obsessive compulsive disorder; Bayer and Sanofi implementing an AI/ML drug discovery method for metabolic diseases [37].

### 2.4. Surgical Robotics 

The surgical robot application is gaining usability in many surgical events relating to orthopedics, clinical neurology, co-clinical oncology, and odontology. SML helps surgeons better access and understand real-time cautions and offer suggestions during the surgical process. The DL-based method could be beneficial in rendering the location and target for the best clinical and surgical practice with better accuracy [38]. However, these AI/SML robots require further authentication to accomplish the best practice. In the future, AI/SML robots will be beneficial for determining the exact amount of blood extraction from blood vessels for report generation and treatments. 

### 2.5. Clinical Trials 

**Figure 4:**
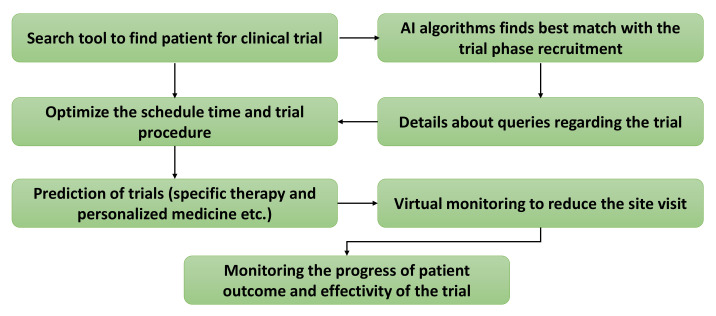
SML-empowered clinical trial procedure. AI = Artificial Intelligence.

After identifying the promising future outcome ahead in SML-based clinical trials, many industries are largely investing in SML-based trials as SML-based implementation makes trial processes faster, well-organized, precise, and more seamless. It was reported that only 13.8% of non-SML-based clinical trials could successfully survive all three stages, although trials using SML have not yet been established [39]. Despite this, they have already been shown to reduce the overall cycle time after SML-based implementation and lower the overall production costs. The SML-based method allows a nonstop data stream from clinicians to be managed, coded, and stored in a database in a structured manner very easily [40]. Details such as EHR, clinical and pre-clinical medical images, and other tests are collected through the practitioner and used to regulate suitable trial methods and techniques. Furthermore, it has become easier for patients to update their health-related information using their wearable SML devices. A step-by-step procedure to automate the clinical trial is described in the following flow chart (shown in Figure 4). 

So far, few SML-based methods have been implemented to monitor patients in clinical trials using audio and visual data. Companies such as AiCure and Brite Health have used this SML technique in clinical trials to regulate the efficiency of the trials and retain patients from dropouts using proper monitoring. Later, the number of patients participating in the trial can be optimized using digital twins by Unlearn.ai, and deep-SML can be used to develop an SML-based patient recruitment system that helps to increase patients’ enrolment in clinical trials. Deep-SML is involved and assists in the analysis of medical records for proper patient identification for the trial [41].

### 2.6. Nanotechnology Research

SML plays a significant role in learning the behavior of nanotechnology and understanding scientific outcomes to pave the approach for the coherent expansion of SML in nano-systems. Currently, SML is used heavily in simulations of nanotechnology systems for many different healthcare application areas [42]. The main work in healthcare is to develop the simulation of how a nanoparticle works and behaves to effectively choose drug carriers, reducing the cost and time of development of nanoparticles. A major challenge of nanomedicine is to determine the effects of different drugs regarding the time, dose, and effectiveness specific to patients. SML can be efficiently integrated with nanomedicine to augment the dose in amalgamation therapy. 

### 2.7. Prediction of Pandemic Outbreak 

In the next few years, one of the most impressive efforts of SML is to forecast an epidemic outbreak based on weather patterns, food habits, genetic populations, pollution, and regional studies. Although there is no method yet developed to show accurate results, the SML method can suggest a solution to bring an outbreak under control, which can help us to act beforehand. One good part of ML is learning the ability to predict the magnitude of an epidemic such as COVID-19 [43]. The SML algorithm helps in imaging or predicting the speed of disease to different sectors by analyzing various structured, semi-structured, and unstructured data from multiple open source and social network data. Cholera and COVID-19 patterns were analyzed during their outbreak in Bangladesh [44] and India using an advanced ML algorithm. 

At the beginning of the COVID-19 pandemic, BlueDot marked clusters of mysterious and uncommon COVID-19 cases that occurred around wet and dry marketplaces in Wuhan. As a result, BlueDot was alerted to the corresponding sectors [45]. An AI engine highlighted a few research papers that reported the probable source of COVID-19 and many cases of pneumonia and flu, with many showing a link with the markets of Wuhan [46]. BlueDot also precisely recognized the cities connected to Wuhan by analyzing the data of international airline ticketing for alert purposes. As predicted by BlueDot, 11 of the top cities were infected with COVID-19 at this time. Many other AI engines have since been developed to predict different outbreaks.

### 2.8. Computer Vision in Precision Medicine and Diagnostics from Medical Imaging

**Figure 5:**
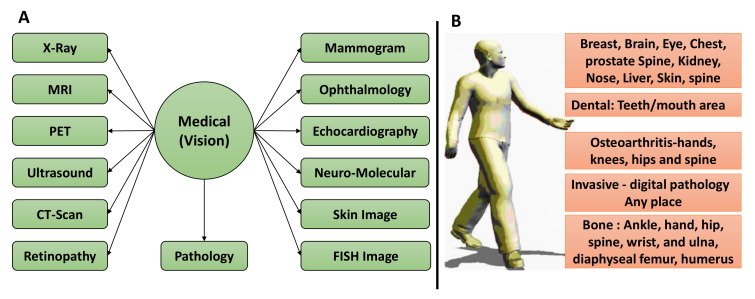
The imaging modality and body parts for ML and CV for clinical studies. (**A**) Different imaging modalities can be used for CV and image processing. (**B**) Imaging from different body parts of humans.

Computer vision (CV) in radiology is expanding and growing its research in the precision medicine and diagnostic area. A recent study [47] extends its use into all other different image modalities, with a particular emphasis on optical coherence tomography (OCT), X-rays, fluorodeoxyglucose (FDG)-positron emission tomography (PET), ultrasound (US), computed tomography (CT), magnetic resonance imaging (MRI), echocardiography, and pathology imaging. The various body part and imaging modality studies are available and are depicted in Figure 5.

In the case of a brain stroke, time is very important [48], and special care and analysis are very necessary for imaging data. As a result, a data-centric model to target lesion identification, classification, and region segmentation (e.g., ventricular) needs to be developed on the collected clinical data [49]. These automatic data-driven applications will allow us to respond quickly in times of crisis events, such as developing and deploying abdomen pain, stroke, and COVID-19 detection models [50]. Other work in medical imaging continues to increase in the field of image translation, reconstruction, quality improvement (e.g., low to high-resolution image enhancement), automatic health report generation, and specific location and lesion tracking over time. In the present scenario, deep learning [51] works efficiently for large data sets and is very useful in diverse tasks, structures, and pathological interpretability. However, interpretability, explainability, selection of correct patient cohort and data preparation, choice of testing criteria, data labeling and reference generation, proper model selection, and performance evaluation need to be strictly monitored despite many claimed successes of deep learning in specific healthcare areas.

Currently, medical professionals mostly rely on a large number of image analyses that obtain the benefits of deep learning-based analysis. One of the biggest and most significant motivational forces for SML, DL, and CV-based methods in clinical non-invasive application is to handle massive amounts of complex unstructured digital data produced from different medical schools and hospitals worldwide. Up until 2020, approximately 20 AI-DL image-based applications [52] were permitted by the FDA USA and the European Union. Now, these are spreading and focusing on multi-modal and multiple modality images, which include X-rays, computed tomography (CT), magnetic resonance imaging (MRI), T1w, T2w, DWI, DCE, FLAIR, ultrasound, and optical coherence tomography (OCT). Most FDA CV/ML approved applications focus on computerized screening, supporting diagnosis, or highlighting the radiologist’s needs. One main progress and success in the study of medical imaging was the detection and recognition of DR, pulmonic embolism, cerebrovascular coincidences, brain injuries, cancer, and chest disease such as pneumonia. Although further complex and novel medical explanations are required, problems typically need empirical thinking using the information of biological procedures. Selectively incorporating meaningful knowledge and information from past studies or patients’ health history could also be beneficial for many cases to reach high accuracy.

### 2.9. Advances in SML-Based Clinical Imaging

**Figure 6:**
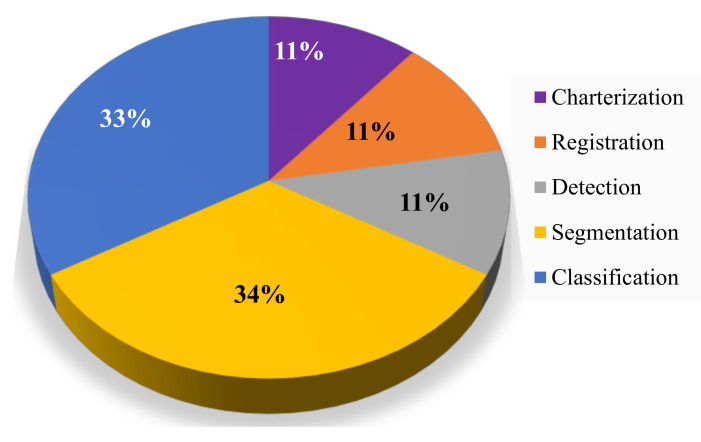
The deep learning application in a medical imaging application.

DL has excellent potential to provide better outcomes with greater availability. Continuation of the current work is needed to establish its use as a proper clinical procedure. The CNN DL method established a recent computerized skin cancer estimation [53]. The authors compared biopsy results, and the algorithm performed even better (>6% improvement) and was consistent concerning dermatologists in three categories, with more than 10% accuracy compared to traditional dermatologists. The performance of the method was inadequate in relation to the levels of accurateness for marking training images. The deep learning application in medical imaging [54] for various avenues (in %) is shown in Figure 6 below. 

### 2.10. Current Stages (in Industry)

The application of CV, ML, and DL in medical imaging is still in its initial stages. Several multidisciplinary research initiatives are taking place among academia, industry, and large corporations. However, apart from specific healthcare and biomedical companies, GoogleBrain [55], DeepMind [56], Microsoft, and IBM are all working on medical imaging technology and SML-based healthcare developments. Many small and medium-sized industries, such as Qure, Prognostic in med, Aidoc, Arterys, Ayasdi, Babylon Healthcare Services, BenevolentAI, Enlitic, TamilNadu government agency, Niramai, Remidio, EnvoiAI, H2O, IDX, MaxQ AI, Mirada Medical, Zebra Medical Vision, Arterys, Gauss Surgical Inc, Zebra Medical Vision, Freenome, Viz, and DiA, are also conducting valuable research. A list of prominent SML methods in healthcare research work and company names are shown in Table 1 below.

The accuracy of the above computer-based works depends on the heterogeneity and amount of data. For clinical-level application programming interface (API) design and software by healthcare businesses, there must be increased collaboration between academia and industry. Collaboration will reduce distance and make it simpler to integrate new ideas with existing solutions by avoiding high expenditures for serious research, extensive data collection and assembly for training and validation, expensive hardware, and developed methodologies for clinical validation.

## 3. A Use Case of New Non-Invasive Diagnostics Development Approaches

Recent studies and research on developing non-invasive imaging diagnostics such as MRI, PET, CT, X-ray, ECG, and many others are slowly being established for screening. Although only a few have some success in clinical trials, invasive coronary angiography (ICA)-based blood flow assessment is one of them. In this section, we discuss the potential of non-invasive imaging tests and computation approaches to reduce the quantity of invasive tests and preserve similar quality in diagnosis for biomedical healthcare with the general and possible vision-based technical approach (CV in medical imaging) as a use case. Traditional SML solution-building, such as pre-processing, detection, segmentation, classification, monitoring, and prediction, is conducted through supervised, semi-supervised, and traditional techniques. Traditional SML solution-building steps are as follows:

Pre-processing: Pre-processing is applied to raw data, signals, or reconstructed radiological images to apply CV techniques for image analysis, data quality enhancement, and data cleaning. For example, deep learning methods are currently used to reconstruct many images from sparse medical (PET\MR) data, low to high-resolution conversion, noise reduction, artifact removal, quality enhancement, and image acquisition.

**Figure 7:**
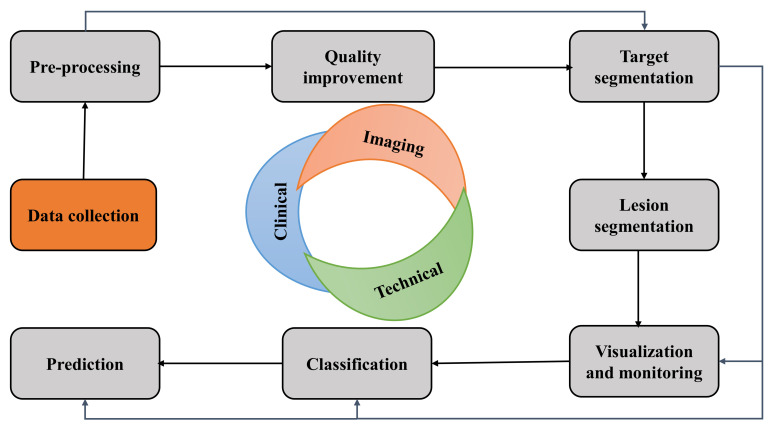
Potential clinical uses of Computer Vision in medical imaging techniques and the corresponding possible tasks.

Detection: Detection highlights a target-specific tissue and region on images likely to contain localized tissue spatial heterogeneity. One example of liver metastases lesion detection and identification of individual lesions with bounding boxes is shown in Figure 7. 

Segmentation: Segmentation delineates the surface area or volume estimation of a target based on intensity, shape, texture, heterogeneity, and edges. One example of the segmentation of a liver metastases lesion outline to extract the largest diameter for follow-up and care of response to therapy of the future liver remnant [57] is shown in Figure 7.

Classification: Classification categorizes the type of irregular lesion from one group to another. For example, liver fat, liver cysts, and hemangiomas can be classified as malignant metastases liver lesions [57]. 

Monitoring: Monitoring denotes the regular follow-up of target lesions to measure changes in position, shape, appearance, and morphology. For example, change in volume, texture, morphology, and intensity in liver metastases and the affected area. 

Prediction selects some features to forestall the progress of ground truth. For example, the prediction of response to therapy or overall survival of liver metastases can be predicted. Figure 2 demonstrates the possible clinical usage of SML methods in liver CT imaging [57]. 

In general, the top-level view of application architecture for a large data set handler includes data collection and annotation, data augmentation, better learning, active learning, semi-supervised learning, transfer learning as a pre-processing, and an intermediate method for the technical aspect. The solution-building tasks and possible technologies are described below.

**Figure 8:**
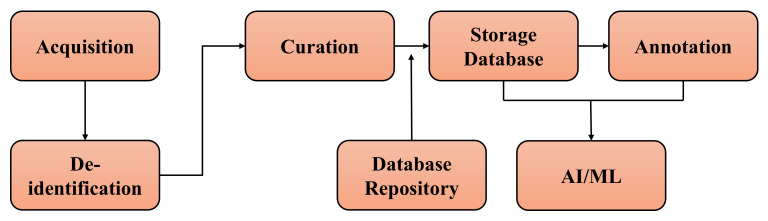
Data preparation pipeline for Supervised Machine Learning solution.

Inference Pipeline: The inference pipeline comprises image processing, outlier detection, and explainability. The image processing pipeline can form a graph, where each node would represent one of the algorithms mentioned in the next section. Based on the use case, this graph will be dynamic and customized (addition and subtraction of nodes). Outlier detection inference pipelines will be paired with a data processing pipeline to better detect out-of-distribution data points to understand input/outlier patterns. Explainable is the technique for visualizing the features that the models in the pipeline are learning and extracting in order to make the solution more comprehensible. One crucial step in the image processing pipeline is the data preparation pipeline. Several key steps for the data preparation procedure [58] are shown in Figure 8, including (i) data acquisition, (ii) de-identification to eliminate private patient information and maintain privacy, (iii) curated data to maintain the imaging and non-imaging quality, (iv) storage database management systems, and finally (v) image labeling and annotation [59].

The technology used to solve traditional solution building steps to handle large amounts of data is written below. 

Data annotation: Automatic or semi-automatic AI tools are required for the annotation of a large number of datasets, which can also be termed as ‘AI helping AI.’ AI helping AI–annotation is essential to control the best practices. Traditional and semi-automatic AI methods, such as active contour, level set, and graph cut, efficiently accelerate the medical image labeling repository at a higher speed and scale. New AI-helping SML annotation methods can reduce the burdens of labeling a high-volume of complex images [60].

Data Augmentation: Data augmentation includes the synthesis of near-to-real data (close to 90%) using a new data generation procedure to build millions of labeled image datasets for detection and segmentation.

Better Learning: To increase the domain adaptation and speed up the learning models, new innovative, weakly supervised, semi-supervised, and self-learning techniques can be very useful. Many researchers use weaker forms of supervision, heuristic generation-based training data, patterns or rules-based implementation, or other classifiers. Weak supervision helps in dealing with noisier inputs from the professional.

Active Learning: The data managing pipeline is significant; automating this management could be beneficial in developing a complete automation system. A never-ending active learning approach to managing a data labeling pipeline with automatic data distribution based on the knowledge derived from domain/models could be very useful for the analysis system.

Semi-supervised learning: A small set of labeled training sets and a larger unlabeled data set can be very useful in the semi-supervised learning environment. The goal is to obtain solid high-level data representation as part of a regularized discriminative model. Recently, researchers developed deep learning algorithms exploiting biophysical models to estimate biological parameters related to human brain neuronal structure and hemodynamic properties [60].

Transfer learning: A pre-trained task-specific model can be transferred to other data sets to fine-tune based on interest.

**Figure 9:**
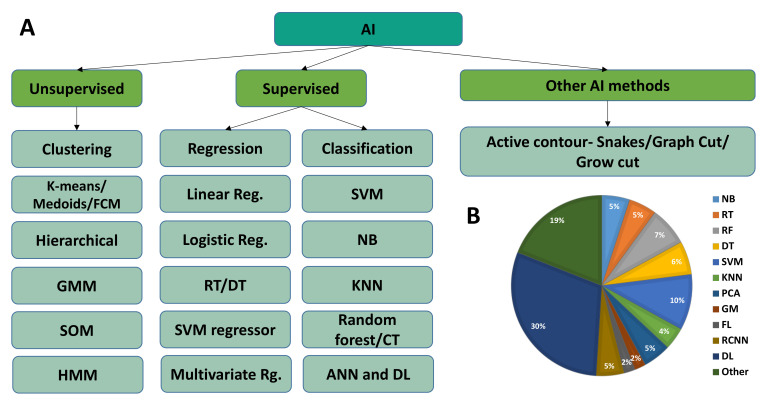
Major AI method used in medical image analysis. (**A**) Method used in image analysis and quantitative medical image data analysis, and (**B**) percentage of each method currently used in medical imaging analysis. Some statistics were also provided by Kumar et al. [1]. The abbreviations used in the figure are as follows: Naive Bayes (NB), support vector machines (SVM), regression tree (RT), random forest (RF), classification tree (CT), classification and regression tree (CART), K-nearest neighbor (KNN), principal component analysis (PCA), hidden Markov model (HMM), Gaussian mixture (GM or GM model), fuzzy logic (FL), regions with CNN (R-CNN), deep learning (DL), self-organized machines (SOM), and artificial neural network (ANN).

There are approximately a dozen ML methods, each captivating a different approach. However, choosing the correct algorithm can seem overwhelming as no decent method fits all considering multiple views. The typical questions and main points to identify the appropriate method are trial and error, the size and type of data, research questions, and the purpose. The commonly used AI in the detection, segmentation, and analysis of medical data is shown in Figure 9. 

Among all of the methods, deep learning is used most commonly [1] in the study of medical data analysis. In general, Python is the most popular programming language used for the deep learning implementation purpose. Most researchers use the common TensorFlow and PyTorch DL libraries for their research. However, many researchers run Keras, Fastai, and Lasagne on top of TensorFlow and Pytorch. 3.

The deep CNN architecture is an advanced image processing and medical analysis method. More explicitly, the CNN layer is used as a building block of most DL architectures for detection, classification, and segmentation based on performance. With more significant computation, CNN can be easily trained on 3D data such as PET, CT, and MRI [57]. The Frequently used deep learning techniques and their use cases [58] in medical data are shown in Table 2.

The choice of the neural network model depends on the application, and the model architecture changes very rapidly to overcome the application problem and generalize the model. ResNet and DenseNet models were identified as very efficient for classification and U-nets for medical imaging-related anomaly identification. Still, feature dependencies were not exhibited competently due to non-optimal discriminative feature association with a semantic class to reach state of the art results. Training and fine-tuning of the hyperparameters of the model using selected metrics are essential to generalize the building blocks of the model to curate the dataset. Table 3 summarizes the appropriate architectures and standards for several medical imaging tasks.

## 4. Comparative Analysis 

The literature study illustrated in Table 4 showcases very brief information such as disease types, data set availability, the method used, and corresponding success. Table 4 will help readers understand the usability of a suitable method for each of the disease types. The research can be used to implement the best-suited method for the detection of various diseases with improved results. 

Table 5 shows the framework of learning-based algorithms, strategies, and the most popular method used in the corresponding fields from the survey and Table 4. The table is divided into three parts: the basic learning style, hybrid learning, and finally, common complex learning strategies that solve several models together [106].

## 5. Challenges from the SML Implementation Side

The challenges towards healthcare transformation in using SML are data issues, data-snake oil, interdisciplinary team building, reproducibility, personalized medicine, moving into clinical practice, data and algorithms, causal AI, product development, and effectiveness and trust in AI-augmented healthcare [107].

### 5.1. Data Issues

Maximum SML-based analytics (especially deep learning) relies on access to large datasets for healthcare data analysis, and all supervised learning requires a labeled training set. Access to high-quality labeled data is crucial and difficult to achieved in the implementation and assessment of SML methods for the co-clinical decision-making process [108]. Creating training labels from known archives data/records requires skilled medical personnel to review patient charts for meaningful label creation. As a result, the time and cost involved in the project increase. On the other hand, many publicly accessible labeled data sets for SML methods are very small in size. Data sets labeled from clinical records can also be used for research, but those are variable in quality, which restricts the efficacy of training in many cases. The performance of the SML/ML depends on the training data, and performance cannot be expected to be more than the training data set will allow. The more quality and heterogeneous data we obtain, the better the data set will perform, and there will be fewer inconsistent images within the training space. 

### 5.2. Data-Snake Oil

The presence of bogus sites and data repositories has increased due to the enormous demand and money floating for data in the healthcare industry. The lack of information regarding the data on those sites and repositories causes trust issues. For example, information concerning patient conditions, symptoms, tests, diagnoses of patients, and treatment possibilities is provided by the American Cancer Society [109] and the Mayo Clinic [110], which are very helpful for further studies. WebMD has been very successful from the beginning of its foundation as a news and information outlet associated with human health providers. WebMD is one of the most visited medical sites in addition to other government/semi-government/private healthcare sites [111]. Similar trustworthy websites and repositories created by experts will help people to direct the proliferation of SML health at all levels. Otherwise, the proliferation of misrepresentation or misinterpretation could obstruct the implementation of AI health. Then, these improper representations or incomplete data may lead to ‘snake oil’ in place of ‘new oil’. Endorsement of best practices and engagement of learned bodies is required to guard against the proliferation of snake oil.

### 5.3. Interdisciplinary Team Building

An interdisciplinary team for building the non-invasive vision aspect of medical image analysis using ML is provided in Table 6.

Multi-disciplinary team building: multi-disciplinary team building is essential in SML for healthcare applications to share their ideas and cooperate with others on joint collaborative work. A project leader is necessary at the top to manage, organize, and preserve the thought of the members of the team in order to guarantee smooth work and flow of the project. Population cohorting: Correct cohort population data selection is required along with catalogue testing that includes all patients. Data with missing values or insufficient studies must be omitted. Data collection and curation: This procedure is usually the most time-intensive stage, but this is very precarious to train for any model. Biobank, images, related information to the data, and radiology reports must be included for any clinical-level AI-based implementation. Data labeling: All lesion or outcome labeling must be conducted by radiologists to train the models. Typical tasks such as annotation, segmentation, ROI drawing, and tracking of targets can be automated using CV techniques. Dataset sampling strategies: The data sets should be independent of each other as much as possible and identically spread for proper implementation in clinical adaptation.

### 5.4. Reproducibility

Healthcare research suffers a lot due to the reproducibility problem, similar to many other areas of science research. Many times, SML-based healthcare models have been verified in unrealistic clinical settings. The repeatability of scanner or any medical device data must be justified with proper statistics before implementing an AI-based method [112]. Many SML methods fail due to bias in the data set, which leads to failures in the generalization of the model. Researchers should use more heterogeneous data and reproduce the same results during their training method. Despite these common setbacks, a few SML-based methods (semi-supervised learning) conserve their performance on new heterogeneous data and show great efficiency in clinical reality [113]. 

The reproducibility crisis leads to massive impediments to translation and an enormous waste of time, effort, money, and faith. Technically isolated positive studies can also fail to convince healthcare practitioners to accept SML health products. Therefore, proper data preparation, reporting criteria, documentation and clarification, development, and updated feature metrics need to be developed to avoid the reproducibility issue [40]. Stepwise documentation will avoid damage to pertinent information and move toward consistent improvement. Honest and repeated rigorous testing of both internal and external validity are essential for the successful deployment of SML in healthcare products at all stages of its implementation process. These developments will lead to imprudent translation.

### 5.5. Personalized Medicine 

SML-based implementation in precision medicine (among many important healthcare applications) is still under-recognized for automation. Many developers are unaware of the inter-disciplinary effort and undervalue the specific challenges of threatening translation. 

Furthermore, confirmatory validation training is required, and it is very costly as sufficient patient enrolment per subcategory is essential. In addition, experts and people familiar with preclinical and clinical implementation and validation studies are required. Projects without high-quality validation and evidence are guaranteed to fail in the actual field. Several cancer research studies have suffered from transformation failures due to a high dependence on low-grade evidence in a small cohort group [114]. Some challenges also translate theoretical fundamentals to practical ones as experience is scarce and the SML method is still in its infancy. Integration of and overlaps of theoretical fundamentals, data science, proper statistical inference, approximation, and attribution, are very active and open research up to questions regarding this type of healthcare research [107]. 

Another real problem is the lack of understanding of the actual meaning of precision medicine, and in many cases, researchers are unable to understand the required level of clinical validation. Proper planning and conceptualization of the project will decrease this failure. Continuous funding with an interdisciplinary team will also provide higher chances of success; excellent and experienced decision-makers will help in the critical assessment of projects.

### 5.6. Moving into Clinical Practice

SML-ML computational-based technologies may be adequately relevant to enhance diagnostic, analysis, and therapeutic procedures, resulting in enhanced results at extremely low costs. For example, in many cases, doctors recommend invasive testing to determine the negative diagnosis result for those patients who underwent serious chest pain. This intrusive procedure is expensive, painful, and time-consuming, which causes patients to become anxious. In many cases, unnecessary use of invasive coronary angiography tests leads to continued levels of high chest pain, where 60% of patients have no other important signs of coronary artery disease [115]. Furthermore, more than 50% of cases would not benefit from revascularization. The diagnosis of coronary artery disease using a computer-aided system will help with cardiac revascularization. 

### 5.7. Use of Hospital Data 

Healthcare institutions often face the challenges of epidemics and the influx of diseases with new/previously unknown and unobserved symptoms. To effectively combat such challenges with lesser fatalities, the interest in advanced technologies has been of utmost importance during recent decades. The applicability of DL and SML to medical research has the potential to solve such crises efficiently. However, data, especially quality data, plays an important role in training and designing such models. The analysis of a large amount of data generated by clinical institutions related to disease symptoms, diagnosis, treatment, digital images, and laboratory analysis results has been proven to provide useful insights into numerous health problems such as is majorly used in the areas of diseases detection and classification, treatment and planning, diagnosis, treatment response prediction, diabetes, cardiology, brain and neurology, retinopathy, pathology image analysis, chatbots, and many more. Clinical data is collected during the ongoing disease investigation of a patient or as part of a formal clinical trial program. Clinical data falls into six major types, namely:

- Electronic health records: Such data is obtained at the point of care at a medical care facility or hospital. These data include administrative and demographic information, diagnosis, treatment, etc. Sites such as NIH and the Stanford center for clinical informatics provide access to such databases as mediated or collaborative access.
- Administrative data: These data are often associated with electronic health records and are reported to a government agency such as the Agency for Healthcare Research and Quality (AHRQ).
- Claims data: These data describe the billable interactions between insured patients and healthcare delivery systems. The sources of claims data can be obtained from the government, such as Medicare, and commercial health firms, such as United Healthcare.
- Patient/disease registries: These data include clinical information systems that track a narrow range of data for specific chronic conditions such as cancer, heart disease, and asthma.
- Health surveys: These consist of data which represents an accurate evaluation of population health and surveys conducted for common chronic conditions.
- Clinical trials, registries, and databases: ClinicalTrials.gov, Cochrane Library, European Union Clinical Trials Database clinical research datasets, The National Heart, Lung, and Blood Institute (NHLBI), Biomedical Translational Research Information System (BTRIS), and the National Institute of Mental Health (NIMH).

### 5.8. Data and Algorithms

Many medical data have the following problems: (a) They are usually broader than their significance. For instance, the data dimension is large, but the sample size is very small. (b) Data sharing and aggregation with other useful information has many legal challenges related to privacy, security, and anonymization. (c) Working on non-established and non-stationarity data is difficult for any supervised method as medical data and diagnostic rules change very frequently over time. (d) During data collection, there may be a large number of missing entries in heterogeneous data sets. (e) Bias plays an important role in the subsequent failures in clinical trials, which is added during the data acquisition stage. Without knowing the proper protocol of data acquisition, it is difficult to translate SML deployment into product development. Sometimes, trials with a limited amount of data lead to inconsistencies in the SML model. Federated learning can be used in those cases where “not enough data” are available [116]. 

Another issue is that the development process must take into consideration the constantly changing protocol and data interpretation standards. The development of SML in medical science is not facilitated by regularly modifying clinical standards. The problem of bias in data collection and the use of SML methods is another challenge to the commercialization of AI in healthcare.

### 5.9. Causal AI

Correct and accurate information extraction is challenging for SML-based healthcare application interfaces. Commonly, SML models initially seem to have impressive performance but successively fail due to unseen data [117]. Many biological issues are changeable and are not just uncorrelated but provide highly unstructured real-world paths. Studying such paths from large heterogeneous data sets requires considerable technical and practical skills. Still, it is sometimes challenging to obtain them from smaller data cohorts. These all lead to a high translation failure rate in clinical trials.

New causal revolution [113] procedures allow straight validation of causal effects through innovative computerized methods. Causality theory, in the procedure of semi-structured learning methods, is one of the best promising tools provide logical support to healthcare SML applications and thus analytically enhance their methodical translation into clinical trials and practice. 

### 5.10. Product Level Development

Despite much progress in digital health technology, SML in healthcare is still very dissimilar to customer tech-based SML. Therefore, transforming the SML in a healthcare project into a real-world application useable product needs an intensive, market-oriented, and specialized attitude to develop a successful product [118].

The industry founders need to raise the fund investment to deploy a consumer SML that is conjugal with the more significant revenue prototypes of SML in health sectors. Therefore, some overall best practice strategies must be followed and documenting the whole process must be from the beginning of the project until the launch of the product, including specific evidence and information on regulatory and development processes. Developed countries such as the USA, the UK, and many European countries follow standard FDA and Johner Institute [119] guidelines for product development. Such standards and professional paths provide guidance and security to all applicable stakeholders for developed software that reduces the failure of SML-based health sector product.

### 5.11. Effectiveness and Trust in AI-Augmented Healthcare 

The real-world use and application of AI in healthcare (especially in clinical practice) are still inadequate despite substantial focus by prominent industry and academy sectors over the last few years [120]. Most of the time, SML is applied to healthcare and implemented successfully without consideration of proper clinical workflows, actual user requirements, trust, safety, and ethical implications. Hence, in building an SML-based healthcare and biomedical system, other essential aspects apart from the medicine and human interaction need to emphasize and improve the adeptness and efficiency of that interaction. Most importantly, healthcare automation must be improved and developed through in-depth knowledge of the interdisciplinary team, centric human-centric understanding, and care pathways.

First, problems must be identified and then the solutions can be designed using the experimental method by consulting with suitable investors, particularly healthcare users and practitioners. Then, an investors/stakeholder team should bring strategic, motivator, leadership, operative, and technical experts to delineate problems, objectives and background, possible success pathways, and intermediate outcomes [14].

Human-centered AI: As an appropriate parameter for the identification of multiple problems, it is important to understand the accessibility and availability of the desired heterogeneous data sets required to build the model and its evaluation. Healthcare using SML systems would function within existing standards and follow them to ensure implementation, providing suitable solutions to the problems and feedback or issues by the end-user to implement appropriate algorithms and update them within the existing workflow. This will help to create a human-centric SML method with the combination of an ethnographic understanding of health systems and a technical point of view of SML.

Experimentation: The implementation of an SML procedure with recurring feedback helps to interpret purpose and envision the uses from the perspective of the end-user and the potential mischief and ethical associations for data security, privacy, and safety. Trying out new thoughts concurrently and learning about the good fitting method is very important in the experiments.

Evaluate and validate: Three-dimensional evaluation and validation (statistical, clinical, and economic utility) are critical factors for healthcare SML where statistical validity includes accuracy, steadfastness, robustness, solidity, and standardization. Then, evaluation of the method in a real-time clinical study on cross-validation, hold-out, and sequential validation is required to determine clinical utility.

Scale and diffuse: Scale-up of the SML-based method requires special consideration for the deployment of different modalities, update of the DL model, monitoring ecosystem, and the disparity between health and biomedical systems.

Screen and monitoring: Continuous monitoring and validation are needed even after any SML method is clinically deployed. It will reduce the risks and adverse events of post-market shadowiness [121].

### 5.12. Precision Medicine 

Precision medicine enables us to tailor healthcare interventions to individuals based on patients’ disease profiles or prognostic information. The treatment considers the genomic variations and a wide range of factors such as age, gender, patients’ immunity and metabolic profile, microbiome, geography, family history, race, and environment vulnerability [122]. Precision medicine is advantageous due to its low healthcare costs, the effectiveness of drug action, and reduced adverse drug response [123]. Precision medicine intends to use patient biology at every stage of treatment instead of population biology.

Curative therapies: Synthetic biology and data preparation have progressed for gene editing and personalized cancer treatments in the last few years. However, the development of this synthetic biology is still extremely incompetent and costly. In the coming days, SML will help us understand, discover, and affect biology with better access to multiple OMICS data. This understanding of biology will also help us increase the adeptness of drug discovery in a much better way to predict the therapeutic agents that anticipate adverse drug effects [14]. The proper implementation of this synthetic biology method will democratize access to new progressive therapies at a meagre cost compared to the original one.

SML-enabled professionals: After a few years, AI/ML will leverage biomedical and health professionals in intensifying the care they provide to patients, allowing them to deliver safe, standard, accurate, and effective care [14]. In the future, it is hoped that clinicians will opt for personalized medicine and use SML-based digital consultation to study the patients’ symptoms and disease using “digital twin” models, allowing effective, safe, and informative testing and enabling the related report to deliver more accurate health check-ups and suggestions from clinicians to patients.

## 6. Explainability in Healthcare

**Figure 10:**
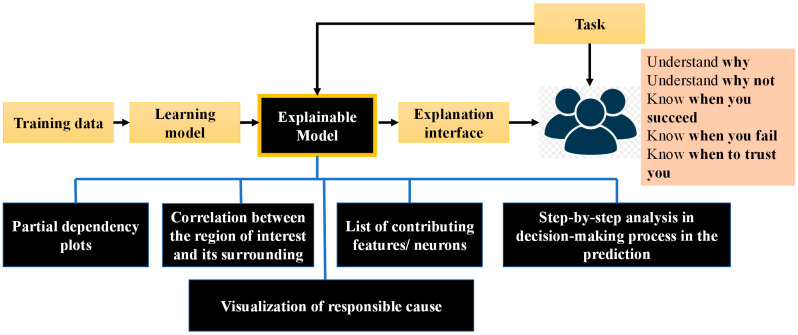
The schematic diagram of XAI for a machine learning-based model to achieve a transparent and trustworthy model.

DL-based SML technology has played an important role in the new age of digital healthcare. Still, the explainability of these models is an unaddressed issue. Explainable SML can play an important role as an auxiliary advancement for potentially overcoming the small sample learning problem by filtering out clinically insignificant information. Furthermore, several high-performance DL algorithms, so-called black boxes, produce results that are incomprehensible to unaided humans. Although these models are capable of outperforming humans in terms of efficiency, it is difficult to provide intuitive interpretations which can validate the findings of the model, define their uncertainties, or derive further clinical understandings from these computational algorithms. With millions of attributes in the DL model, understanding what the machine sees in clinical data, such as radiographical images and dermatoscopic images, can indeed be very difficult [124,125]. It is important to show that a high-performance DL model properly recognizes the relevant portion of the image and fails to overemphasize irrelevant data (See Figure 10). Recent studies have started to understand what these black boxes are through visualization methods. Occlusion maps [60], salience maps [126], class activation maps [127], and attention maps [128] are some often used levers. Since the outcome is a radiographic image, localization and segmentation techniques may be more easily interpreted. Model interpretation, on the other hand, remains significantly more challenging for DNN models trained on non-imaging data other than images, which is now a common problem for continuing research attempts.

In healthcare, DL-based SML technology has become very popular for diagnosis [129], prognosis, treatment planning, and patient management. Many unresolved topics in the medical profession have sparked clinical studies utilizing deep learning and SML techniques. However, in the medical sector, the challenge of understandability is still in its early stages. More specifically, interpretability in clinical sectors includes issues that are not recognized in other fields, such as risk and obligations. It can even cost a life if medical analysis and critical decisions are made without the explainabilities of these SML models. In addition to these legal considerations, it is a critical problem that might have disastrous consequences if used maliciously.

As a result, recent studies [130] have started to focus on the explainability of these black boxes in the medical domain. More precisely, researchers now focus more on understanding the model, its explainability, and the importance of understanding in the medical field. Many researchers have followed the logical XAI strategy of providing interpretability to their predictive algorithms. These strategies rely mostly on ensuring the interpretability of simpler SML models while boosting their performance through refinement and optimization techniques. The different modules, including the interface and its requirements of XAI to achieve a trustworthy healthcare automation model, are shown in Figure 10. 

## 7. Ethical Implications 

Many researchers encounter various medical, occupational, technological, and ethical modifications regarding SML development in the healthcare field. Governmental, semi-governmental, and other controlling bodies should limit negative implications to create structures for proper monitoring of critical issues and responsibilities. Another challenging issue for our novel technology in the healthcare industry is transparency. It is difficult to interpret some medical imaging and analysis with the most advanced algorithms, such as deep learning [131]. This makes us unable to answer the question ‘why’, which is very important in the healthcare and biomedical industry. Medical practitioners may be unable to provide an explanation even if they are familiar with their operation. Another issue is failure. It can be difficult to establish accountability for the medical practitioner if the system mistakes in the patient diagnosis and treatment [122]. Many times, SML/ML methods in biomedical and healthcare may have a biased decision based on data collection, for example, gender bias due to the training data when those are not causal factors [123]. Finally, the use of smart devices and software to help human efficiency increases transparency, responsibility, security, and privacy issues. 

## 8. Radiomics

Radiomics is a branch of medicine that uses data characterization algorithms to extract a large number of quantitative features from medical images. The data are analyzed in order to improve decision support. It has the potential to reveal disease characteristics that are difficult to detect using only human vision. Radiomics is a technique that retrieves a significant number of specific quantitative characteristics from radiographic images that go beyond the degree of precision visible to the natural visual system. X-rays are noninvasive imaging procedures which are carried out to detect fractures, artifacts such as catheters and stents, and diseases such as COVID-19 and pneumonia. On the other hand, CT scans provide extra pixel information, which includes the attenuation coefficient of individual pixels. As a result, selective information regarding target lesions can be specifically viewed. However, 3D imaging techniques such as MRI and PET provide detailed special information regarding highly metabolic regions. Since these techniques involve the injection of certain radioactive elements such as FGD, tissues with high metabolic rates are highlighted. This helps in the easier detection of cancer cells [132]. These features can be better explained with software such as Pyradiomics [133] and LifeX [134]. Artificial intelligence, particularly deep learning, analyzes and learns from the image frame sequence entirely; however, radiomics specifies only the contour of a specific disorder. As a result, we believe that DL and radiomics provide distinct imaging indicators. However, the performance of radiomics is more robust in the case of small data. Therefore, in order to avoid overfitting in DL models, these features should be included. 

## 9. The Future of SML in Biomedical and Healthcare

The main challenge for AI/ML in biomedical and health care is not only developing novel methods that are beneficial but also ensuring their acceptance in real-world clinical practice on a daily basis. SML and ML must be approved by a regulatory body, combined with EHR systems, standardized, proper training and taught to medical practitioners, and most importantly, updated over time for extensive adoption. We expect that SML will master providing diagnosis and treatment recommendations based on the imaging in the near future. Given the quick developments in SML for medical imaging examination, it seems possible that radiologists and pathologists will prefer automatic image analysis by the computerized method. Automatic speech and text recognition will be engaged for patient communication and to collect notes of clinical use. It is very clear that SML-based software will not replace human clinicians but rather help them on a bigger scale and increase their judgment for patient care. Over time, clinicians will move toward job scheduling and draw on exceptional human assistance such as responsiveness, encountering new cases, encouragement, and integration of the big picture. However, lower-level healthcare practitioners may lose their jobs in the future if they are not willing to update their work alongside SML [124].

In healthcare, SML has already started changing the patient experience, research on clinician medicine practice, and how the drug discovery industry operates. By 2030, SML will have accessed many data sources to diagnose and predict disease more accurately, identify illness trends, and improve therapy and care. A person’s risk of developing certainn illnesses and recommending a path to avoid those could be conducted by an SML-based system. Patient waiting times in hospitals and health systems will be reduced using SML-based systems, and that will increase the efficiency of the hospital management system. Mobile application-based SML will occur in radiology, drug discovery, patient risk identification, and primary care areas. 

This study is basically an overview of the use of artificial intelligence in the healthcare sector, and we have not covered techniques such as federated learning, optimization of learning function [135], unsupervised learning, semi-supervised learning, weakly supervised learning, zero-shot learning, and federated and fin grained learning in detail, which can be the potential limitation of this study and the future scope of schematic review.

## 10. Conclusions and Discussion 

We are at a turning point of convergence of traditional healthcare practice to technological application, and it needs to overcome issues of real-world clinical trials. Higher accuracy and reduced time are critical for effective planning and treatment when it comes to efficient diagnostic care. SML is a massive and diverse dominion of data, processes, analytics, neural networks, deep neural networks, and visualization techniques that are continuously increasing and updating the requirements of biomedical and health sectors efficiently and accurately. Industrial SML in disease diagnosis could further extend to Alzheimer’s disease, cancer and its biology, diabetes and kidney-related disease, chronic illnesses, heart-related disease, bone-related, fatal brain, stroke, and cerebrovascular-related issues, high blood pressure, skin-related problems, and liver metastasis in the next five years. The clinical diagnostics and decision-making problem must be solved on a prior basis to resolve and constantly improve our ability to treat diseases more accurately and effectively, although few reported advancements have occurred over the past few years.

The blurriness between end-users and SML-implemented methods must be removed from time to time in order to build trust in AI/ML software used in the biomedical and healthcare sectors. The methods’ expandability should be investigated further. Many works are required to train the SML-based model, which can then be fitted to other methods, such as transfer learning, to increase the accuracy of prediction for automated diagnosis. In the future, the flaws and gaps (if any) in SML methods, as well as the overall SML techniques, must provide an equally valuable and understandable relationship between medical users and SML application developers. Furthermore, a distributed federated, semi-supervised, self-supervised learning model can be used to create a solo training model that will aid in the timely diagnosis of diseases in remote and rural villages. The enlargement of translational research and development needs to be conducted with proper care to build lab-level implementation to product development. Alongside this, we require proper funds and investment to upskill the healthcare workforce and to understand the perspective and potential of the SML-enabled healthcare system. The advanced form of AI/ML and data science should have the ability to update very quickly and combine with a small device to achieve high accuracy in personalized, predictive, and portable healthcare systems. However, the procedures for data access ethics, security, privacy and ML implementation, evaluation, validation, and adoption must be taken care of during implementation. It is also critical to build ‘trusted’ SML algorithms that can be embedded into suitable systems.

It is expected that the next 10 years will subsequently create great data medical sets and novel SML tools that will provide insight and value to society by translating better clinical outcomes and solving the major issues and problems of today.

## Figures and Tables

**Table 1:** Prominent work around the world in industry and company related to CV in medical imaging.

Industry/Company Name	Task Performed
Google Brain	DR screening solution and also working on AI to improve breast cancer detection
DeepMind	Diagnosis and referral in retinal disease
Microsoft	Optimization of cancer treatment and radiotherapy-based planning
IBM	AI in healthcare and life sciences (e.g., predict the specificity of T cell receptors)
Qure	AI-assisted chest X-ray solution
In med prognostic	Volumetric analysis of the brain using AI
Tamil nadu e govt agency	AI-based cataract detection system
Niramai Health analytix	Breast cancer screening solution
Remidio	DR screening tool
Arterys	Proper blood flow visualization and quantification from MRI of whole-body images
Gauss Surgical Inc	Real-time tracking and monitoring of blood loss after surgery
Zebra Medical Vision	AI chest X-ray triage
Freenome	Cancer detection from blood cells imaging
Viz	Early signs of stroke prediction and detection
DiA Imaging	AI-powered ultrasound image analysis solution
RetinAi	“Discovery Platform” Glaucoma, DR, and macular degeneration (MR) data collection and analysis
BrainMiner	AI-based brain MRI scan analyzing systems
Lunit	AI-based automated chest X-ray interpretation

**Table 2:** Frequently used deep learning techniques and their uses.

Technique	Advantage	Disadvantage
Deep Neural Network (DNN): Minimum two layers, many complex nonlinear associations among the layers. Mainly used for regression and classification purpose.	High accuracy	The very slow learning process. Nontrivial training procedure due to back propagation to the aforementioned layer.
Convolutional Neural Network (CNN): Transformation of 2D to 3D convolutional filters, and also works well for 2D data.	High accuracy on 2D data and fast learning model.	Need lots of labeled data for perdition.
Recurrent Neural Network (RNN): Weight sharing across all neurons and steps and sequential learning.	Provides high accuracy for visualization aspect of medical image analytics problems.	Many issues due to gradient vanishing and need a large amount of data.
Deep Boltzmann machine (DBM): It consists of unidirectional linking between all hidden layers.	More robust inference as top-down feedback incorporates.	Parameter optimization is not promising for large data.
Deep Belief Network (DBN): The greedy approach used in each layer. Unidirectional association between two layers at the top of layers.	Work well for similar data.	Training is expansive due to initialization problems.
Deep Autoencoder (DAE): The no. of I/P is equal to the number of O/P., reduced dimensionality.	Labeled data is not needed.	Training may suffer due to convergence and vanishing problems.

**Table 3:** General structures and common performance measurement metrics for various medical imaging-related tasks performed using SML methods.

	Detection	Segmentation	Classification	Prediction
Features	Using various shapes such as circles, rectangles, and squares and labels using different binary masks, overlays, etc.	Texture, shape, position, and intensity Higher order features	Texture patches 3D Radiomics features Morphological features Imaging metrics	Texture and shape Survival time Dynamic modeling Imaging metrics
Model architectures	CNN	UNet/Unet++	Dense net	Efficient net/CNN
Performance metrics	Mean average precision or mAP Intersection over union (IoU)	Mainly IoU and F measure, precision, recall, relative area error, and accuracy	Area under the curve (AUC) or receiver operating characteristic (ROC) Confusion matrix	AUC/ROC Confusion matrix Coefficient of determination

**Table 4:** Reported success and comparative analysis for the detection of different diseases.

SL. NO.	Authors	Application	Dataset	Method Used	Reported Success
1	Murat SeçkinAyhan et al. [61]	Ophthalmology	Kaggle, Asia Pacific Tele-Ophthalmology Society, Messidor and Indian Diabetic, Retinopathy Image Dataset (IDRiD)	Deep neural networks (DNNs)	Accuracy ≥ 90%
2	Elineide S. dos Santos. et al. [62]	Skin cancerdermoscopic images	PH and DermIS datasets	Linear iterative clustering (SLIC0) algorithm	Accuracy = 96.78%
3	Ghorbani, A et al. [63]	Interpretation of echocardiograms from heart	The Stanford Echocardiography Database	CNN- EchoNet	Area under the curve = 0.89
4	Bulten, W. et al. [64]	Grading and diagnosis of prostate cancer	Data from Radboud University Medical Center and Karolinska Institutet for PANDA challenge and competition	Multiple AI and machine learning methods	Error or miss: 1% for cancer and 1.8% for the pathologist
5	Rakesh Kumar Patnaik et al. [65]	Prediction of liver function from quick breath monitoring	A pilot study at a local university	Different regression and support vector machines (SVM) and various forms of decision trees	Correlation *p* < 0.01 between unhealthy and healthy samples
6	S Roy et al. [40]	Treatment response prediction of triple negative breast cancer—Co clinical trial (PET)	WashU medical school-imaging facilities	Radiomics, support vector machine, and naïve Bayes	Accuracy ≥ 72%
7	K Dutta et al. [66]	Preclinical breast cancer segmentation (MRI)	WashU medical school-imaging facilities	Dense UNet deep network	F-measure ≥ 94.8%
8	I. Anand et al. [67]	Breast tumor segmentation from magnetic resonance images (MRI)	RIDER breast MRI dataset	ResU-Net Architect	Accuracy = 73.22% and dice coefficient = 85.32%
9	S Roy et al. [68]	Preclinical breast cancer optimal radiomics (MRI)	WashU medical school-imaging facilities	Statistics and radiomics	SNR between 28 to 33/NA
10	Varadarajan et al. [69]	Edema grades of diabetic macular from fundus image	Rajavithi Hospital clinical data from 2010 to 2018	Deep neural network	Area under the curve = 0.89
11	Chetty, G et al. [70]	Tumor lesion segmentation from brain MRI	BraTS open challenge data set, 2018	Modified UNET architecture	Dice score = 94%
12	M A. Savaikar et al. [71]	Prediction of response to carboplatin therapy for triple negative breast cancer (TNBC) from mice PET images	WashU medical school-imaging facilities	Change in imaging metrics a standard uptake value	F score of SUVmax = 73% SUV25 = 72% SUV(SS) = 74%
13	Ching-WeiWang et al. [72]	Segmentation and classification of bone marrow from Hematopathology image	Own institute dataset	Hierarchical deep learning framework	recall and accuracy of 0.905 ± 0.078 and 0.989 ± 0.006
14	Liu, M. et al. [73]	Detect marginal bone loss around implants	Peking University School and Hospital of Stomatology	RCNN or region-based CNN architecture	Sensitivity 81%
15	Lea, W.Wi et al. [74]	Bone-age software on real-world data	Home hospital-pediatric clinic	Deep learning (DL)–based software	Correlation = 98% and *p* ≤ 0.025
16	A Mitra et al. [75]	Glaucoma detection and analysis from retinal fundus image	ESSIDOR and Kaggle datasets	Deep Convolution Neural Network (CNN)	Accuracy was 99% on both two datasets
17	Mall, P.K et al. [76]	Musculoskeletal radiographs, X-ray images	Musculoskeletal radiograph (MURA)	Combination of ChampNet with CLAHE and other types	Highest accuracy was 96%
18	S Roy et al. [77]	Brain abnormality segmentation from MRI of brain images	Brainweb database	Iterative Level Set	Accuracy ≥ 75%
19	S Roy et al. [78]	Multiple small target lesion detection and segmentation from MRI of the brain	Harvard brain atlas network	Hybrid level set and thresholding	correct detection ration = 92.6%
20	Guan B et al. [79]	Predict pain progression in knee osteoarthritis	University of Wisconsin	deep learning (DL)	Area under the curve = 0.77
21	Iizuka, O. et al. [80]	Detection and classification of gastric and colonic epithelial tumors from histopathological slides	The Cancer Genome Atlas open dataset	CNNs and RCNN (recurrent neural net.)	Area under the curves (AUC) was 0.97 and 0.99
22	Abbas, A. et al. [81]	COVID-19 detection classification from chest X-ray	Images were collected from several hospitals around the world	Decompose, Transfer, and Compose based on deep CNN	Accuracy of 93.1% (with a sensitivity of 100%)
23	Wulczyn E et al. [82]	Multiple cancer types from histopathology images	The Cancer Genome Atlas open dataset	Multiple deep CNN modules	Disease specific survival prediction was significant *p* < 0.0001
24	Sabanayagam C et al. [83]	Chronic kidney disease prediction from retinal images	Institute data-Singapore Eye hospital	Deep learning algorithm (CNN)	AUC was 0·88 and 0·71 in internal and external validation
25	Zhang, Y. et al. [84]	Classifying endometrial lesions	Hengjing Hospital of China Medical University	Tuned VGGNet-16 model	Accuracy = 91%
26	Pierre Pinochet et al. [85]	Suspected cancer location detection from PET and computed tomography (CT) images	Clinical data from the Henri Becquerel Cancer Center	CNNs	Dice score was 65%
27	May Sadik et al. [86]	Bone marrow uptake estimation and detection in Hodgkin’s lymphoma patients from PET images	Sahlgrenska University Hospital	CNN	Sensitivity = 65% and Specificity = 98%.
28	Janani Venugopalan et al. [87]	Early prediction of Alzheimer’s disease stage from MRI images	ADNI-Alzheimer’s Disease Neuroimaging Initiative open database	Novel deep learning and multi-modality data	accuracy of 0.75 ± 0.11
29	Esraa A. Mohamed et al. [88]	Breast cancer detection system from thermograms	DMR-IR real data	U-Net network and world-class deep learning model	Accuracy = 99%,
30	Muhammad TariqSadiq et al. [89]	EEG-based robust brain–computer interface framework	--	Pre-trained CNN models	average classification accuracy of 99.52%
31	Rodolfo M. Pereira et al. [90]	Analysis of chest X-Ray and identification of COVID-19 from X-ray	RYDLS-20	Pre-trained CNN model	F1-Score of 0.89
32	Alexandre Bailly et al. [91]	Performance prediction of various machine learning methods on simulated data	Simulated data	Deep neural network and logistic regression	AUC = 0.80 and 0.85 for neural net and penalized regression.
33	Raquel Sánchez-Cauce et al. [92]	Cancer localization from breast thermal image	DMR database	Deep CNN architecture	97% accuracy
34	Wallis, D. et al. [93]	Identification and localization of lymph nodes from pathological mediastinal	Clinical data obtained by the authors	U-Net and 3D CNN	Sensitivity = 87%
35	Johnsson, K. et al. [94]	Standard report generation and automated annotation from PET imaging data	Progenics Pharmaceuticals, Inc., USA	Deep learning	Dice scores ≥ 0.79.
36	Capobianco, N. et al. [95]	Whole body PET uptake and prostate cancer grading classification	Institute data-Technical University of Munich	Deep learning methods	80.4% average precision
37	Etminani, K. et al. [96]	Alzheimer's and dementia prediction and diagnosis of cognitive impairment	European DLB (EDLB) Consortium	3D deep learning model	AUC = 0.96
38	Mehranian, A. et al. [97]	Whole-body PET oncology scans quality enhancement and noise reduction	Hospitals-Oxford University	Deep learning	Scan time reduction by 50%
39	Xue, S. et al. [98]	Low to standard dose in PET imaging quality conversion	Harvard Medical School and Massachusetts General Hospital	Cross-scanner and tracer-based deep neural network	Conversion significantly *p* < 0.05 and normalized dose acquisition *p* < 0.05
40	Wang, L. et al. [99]	Differentiating between nontuberculous mycobacteria and Mycobacterium tuberculosis from a chest CT image	Data was collected from 2014 to 2020 from Tianjin Haihe Hospital	deep learning framework (3D-ResNet)	AUCs = 0.86
41	Merali, Z et al. [100]	Spinal cord compression identification and detection from cervical MRI scans	University of Toronto-Home institute data	CNN model	AUC = 0.94
42	Awan, M.J. et al. [101]	Detection of anterior cruciate ligament injury from MRI scans using deep learning	Clinical Hospital Centre Rijeka	ResNet-14	AUC = 0.99
43	Awan, M.J. et al. [102]	Comparative analysis of machine learning models to identify the condition of three anterior cruciate ligament tears	Clinical Hospital Centre Rijeka	Random forest, categorical boosting, light gradient boosting machines, and highly randomized classifier	AUC = 0.99
44	Awan, M.J. et al. [103]	Automatic segmentation of the anterior cruciate ligament tears from MRI	Clinical Hospital Center in Rijeka, Croatia	U-Net	Accuracy = 98.4
45	Saeedizadeh N. et al. [104]	A segmentation framework to detect chest regions in CT images which are infected by COVID-19	COVID-19 CT segmentation dataset	U-Net (COVID-19 TV-U-Net)	Dice score = 86%
46	Stefano, A. et al. [105]	Automated identification and segmentation of COVID-19-infected regions using CT	COVID-19 Lung CT Lesion Segmentation Challenge—2020 (COVID-19-20)	Deep learning framework (C-ENET)	Dice score = 75%

**Table 5:** Learning outlines and approaches with some popular techniques in the field of medical imaging.

Learning Type	Methods	Applications
Basic learning frameworks		
Supervised learning	Different types of classification and regression trees (CART), RF, NB, SVM, ANNs, and RNNs	- Disease detection and diagnosis - Target lesions segmentation - Radiotherapy dose estimation - Multimodalities imaging and synthetic image generation
Unsupervised learning	Dimensionality reduction (e.g., PCA, LDA), different clustering—unsupervised (e.g., K-medoids, K-means), and auto encoders	- Classification of the patient population - Different domain adaptation tasks
Reinforcement learning	Markov model and reinforcement Q-learning	- target lesion separation and image reconstruction - Treatment response prediction and growth prediction
Hybrid learning frameworks		
Semi-supervised learning	Generative adversarial networks	- Abnormality classification - Synthetic image creation - missing data handler
Self-supervised learning	Augmentation, texture features, patch extraction, and rotation	- Target lesion classification, detection, and/or segmentation
Complex learning strategies		
Transfer learning	Causative, perspiration, and unsupervised	- Toxicity estimate of radiotherapy dose - Clinical practices adaptation - Model simplification
Ensemble learning	Bagging bootstrap aggregating and boosting (e.g., AdaBoost, gradient boosting)	- Prediction of radiotherapy dose - Uncertainty Estimation - Stratification of patients

**Table 6:** Checklist of steps required for project management involving CV and DL.

Task/Type	Management
Team	❏ Team lead and principal investigator (e.g., faculty or industry expert in the related fields). ❏ Domain experts (e.g., doctors, medical practitioners, surgeons, radiologists, hematologists, and pathologists). ❏ Imaging analyst (e.g., image scientist, radiologist, researcher, or data scientist). ❏ Technical researchers (e.g., researchers, AI/ML/data scientists, and imaging experts).
Cohorting	❏ Selection process (e.g., by target population, heterogeneous data, or database). ❏ Identification of data source (clinical and open-source data).
Data	❏ Security and privacy. ❏ Collection and cleaning. ❏ Examination and quality controller. ❏ Annotations and data labeling. ❏ Reference data generation. ❏ Training and test division.
Model	❏ Development of a new model. ❏ Proper model selection. ❏ Hyper parameters fine-tuning. ❏ Test on an unconnected dataset. ❏ Performance measurements.
Hardware	❏ Define the best memory machine/system. ❏ Central processing unit and graphics processing unit configuration.
Regulatory	❏ Market research to commercialize. ❏ Feature management system.
Clinical adoption	❏ Validate clinically and measure the performance. ❏ Deployment for clinical practice.

## Data Availability

Not applicable.
